# Relative strain is a novel predictor of aneurysmal degeneration of the thoracic aorta: An ex vivo mechanical study

**DOI:** 10.1016/j.jvssci.2021.08.003

**Published:** 2021-10-08

**Authors:** Peter Chiu, Hong-Pyo Lee, Alex R. Dalal, Tiffany Koyano, Marie Nguyen, Andrew J. Connolly, Ovijit Chaudhuri, Michael P. Fischbein

**Affiliations:** a Department of Cardiothoracic Surgery, Stanford University School of Medicine, Stanford University, Stanford, Calif; b Department of Mechanical Engineering, Stanford University, Stanford, Calif; c Department of Pathology, University of California San Francisco, San Francisco, Calif

**Keywords:** Thoracic aortic aneurysm, Elastic modulus, Fracture toughness, Relative strain, Machine learning

## Abstract

**Objective:**

Current guidelines for prophylactic replacement of the thoracic aorta, primarily based on size alone, may not be adequate in identifying patients at risk for either progression of disease or aortic catastrophe. We undertook the current study to determine whether the mechanical properties of the aorta might be able to predict aneurysmal dilatation of the aorta using a clinical database and benchtop mechanical testing of human aortic tissue.

**Methods:**

Using over 400 samples from 31 patients, mechanical properties were studied in (a) normal aorta and then (b) between normal and diseased aorta using linear mixed-effects models. A machine learning technique was used to predict aortic growth rate over time using mechanical properties and baseline clinical characteristics.

**Results:**

Healthy aortic tissue under in vivo loading conditions, after accounting for aortic segment location, had lower longitudinal elastic modulus compared with circumferential elastic modulus: −166.8 kPa (95% confidence interval [CI]: −210.8 to −122.7, *P* < .001). Fracture toughness was also lower in the longitudinal vs circumferential direction: −201.2 J/m^3^ (95% CI: −272.9 to −129.5, *P* < .001). Finally, relative strain was lower in the longitudinal direction compared with the circumferential direction: −0.01 (95% CI: −0.02 to −0.004, *P* = .002). Patients with diseased aorta, after accounting for segment location and sample direction, had decreased toughness compared with normal aorta, −431.7 J/m^3^ (95% CI: −628.6 to −234.8, *P* < .001), and increased relative strain, 0.09 (95% CI: 0.04 to 0.14, *P* = .003).

**Conclusions:**

Increasing relative strain was identified as a novel independent predictor of aneurysmal degeneration. Noninvasive measurement of relative strain may aid in the identification and monitoring of patients at risk for aneurysmal degeneration. (JVS–Vascular Science 2021;2:1-12.)

**Clinical Relevance:**

Aortic aneurysm surveillance and prophylactic surgical recommendations are based on computed tomographic angiogram aortic dimensions and growth rate measurements. However, aortic catastrophes may occur at small sizes, confounding current risk stratification models. Herein, we report that increasing aortic relative strain, that is, greater distensibility, is associated with growth over time, thus potentially identifying patients at risk for dissection/rupture.

**Article Highlights**

• **Type of Research:** Human study
• **Key Findings:** A linear mixed-effects model compared the mechanical properties of over 400 samples from 14 diseased and 17 control thoracic aortas. Diseased aorta revealed decreased toughness, −431.7 J/m^3^ (*P* < .001) and increased relative strain, 0.09 (*P* = .003), compared with control aorta.
• **Take Home Message:** Relative strain is a novel independent predictor of aneurysmal progression and may aid in identifying and monitoring patients at risk for aneurysmal progression.

Thoracic aortic aneurysms have an incidence rate of approximately 10.4 per 100,000 person-years.^1^ With aneurysmal progression, patients face increasing risk for life-threatening aortic complications.^2^ Current guidelines for replacement of the aorta are based on aortic size, growth rate, symptoms, and the presence (or absence) of connective tissue disease.^3^ However, aortic catastrophes may occur at smaller sizes.^2^^,^^4^ Moreover, aortic growth rate requires multiple repeat studies over time leading to potentially significant lag time in addressing at-risk aneurysms. The decision to operate on a patient with a thoracic aortic aneurysm must balance the risk of aortic catastrophe against the risks associated with the operation. Additional means for risk stratification would greatly improve the care of these patients.

Pichamuthu et al^5^ observed that ascending aortic tensile strength may vary greatly between aneurysms of similar size depending on the presence or absence of a bicuspid aortic valve further reinforcing that maximal aortic diameter may be insufficient to appropriately assess the risk of watchful waiting as compared with surgical intervention. Martin et al^6^ suggested that patients with stiffer aortic aneurysm tissue may be at increased risk for dissection and rupture and proposed that decreased tissue compliance be used as an alternative to maximum aortic diameter. Sommer et al^7^ further delineated triaxial shear data and uniaxial extension data that demonstrated differences in tensile strength when comparing the circumferential, longitudinal, and radial directions potentially allowing for the development of three-dimensional failure models that may provide a more realistic assessment of failure risk than absolute size and growth. Despite the interesting findings reported in the literature, bench-top failure models may not be able to accurately assess the risk of aortic rupture as the outcome of interest, that is, cannot be observed and validated in vivo. Furthermore, these bench top models offer only a snapshot in time and do not account for progression of disease with the concomitant changes in mechanical properties over time, that is, the mechanical properties of the aorta during the tests may not be representative of the properties at the time of aortic catastrophe.

With the development of noninvasive methods to measure mechanical behaviors, including computed tomography (CT) and transesophageal echocardiogram,^8^, ^9^, ^10^ the possibility of using metrics other than either maximum aortic diameter or aortic growth rate is becoming increasingly feasible. Guo et al^10^ and de Beaufort et al^8^ were able to demonstrate that gated multidetector CT angiography may be able to determine aortic strain in both the longitudinal and circumferential directions. Liu et al^11^ further correlated in vivo estimates of material properties with experimental data from planar biaxial tests in two patients, suggesting that noninvasive studies may be able to correlate with benchtop testing. Given the emergence of these noninvasive techniques and the increasing efforts to these modalities with benchtop testing, we undertook the current study to determine whether the mechanical properties of the aorta might be able to predict aneurysmal dilatation of the aorta using a clinical database and benchtop mechanical testing of human aortic tissue.

## Methods

### Tissue collection and patient selection

After approval from the institutional review board at Stanford University, diseased tissue was obtained from patients scheduled for elective aortic repair at Stanford Healthcare between March 2016 and February 2017. After obtaining informed consent for research from each patient, tissue was collected from the operating room. Control tissue was obtained from both heart transplant recipients and unused donor tissue. Among heart transplant recipients, only first-time sternotomy heart transplant recipients with normal aortic dimensions (aortic root and ascending aorta) were included in the study. Unused donor tissue (aortic root, ascending, arch, or descending thoracic aorta) was obtained after verifying research consent.

Samples were stored on ice for transport and kept in a +4°C refrigerator for a total duration of no more than 24 hours of ischemic time, that is, time from the aortic cross-clamp. All samples were tested fresh. Although there has been some evidence that suggests that freezing does not affect the mechanical properties of arterial tissues,^12^ this result has not been shown consistently.^13^, ^14^, ^15^, ^16^

### Biomechanical testing

Perivascular fat was removed from harvested tissues without removing the adventitia. The samples were then cut along both the longitudinal, that is, along the length of the aorta from proximal to distal, and circumferential directions, that is, at a single level in a ring around the aorta, and more than three rectangular specimens were taken out from each directional tissue. For each specimen, thickness and width were measured with digital calipers at three different points. Representative values were calculated by averaging these measurements. The specimens were preserved in buffer solution until testing to prevent dehydration. Mechanical tests were performed within 24 hours after explant. Before mechanical testing, surrounding adipose, connective tissues, and blood clots were removed from the adventitia and the samples were washed three times in Dulbecco's phosphate-buffered saline.

**Fig 1:**
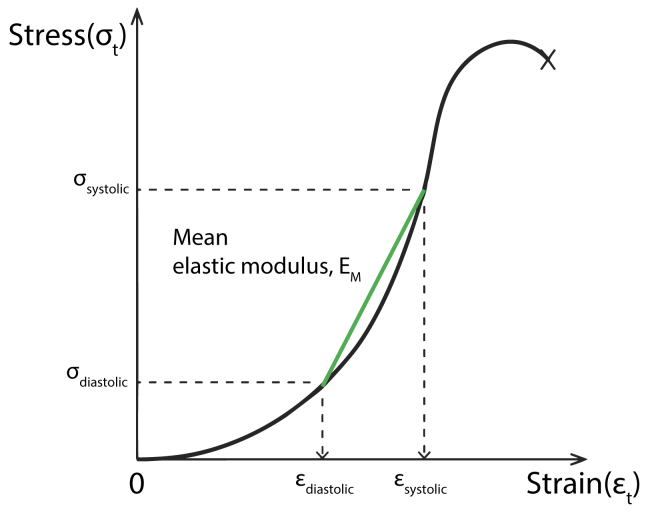
Method for calculating aortic tissue elastic modulus using experimentally derived stress-strain curves. Diastolic and systolic stress (σ_diastolic_, σ_systolic_) and strain (*ϵ*_diastolic_, *ϵ*_systolic_) are plotted to demonstrate the calculation of mean elastic modulus *(E*_M_).

A uniaxial tension test was conducted with a tensile testing machine (Instron 3342; Instron, Norwood, Mass) with a 100 N load cell. Cross-hatched clamps were used to grip each specimen in order to prevent slippage during the test and avoid damaging samples. The gauge length between the ends of the clamps was ensured with more than twofold of the width of the specimen to prevent the shear deformation and local narrowing of the sample due to the gripping.^17^ The initial length of the specimen between the ends of the clamps was measured three times with digital calipers, and values were averaged to calculate the initial length of the specimen. Specimens were not preloaded and preconditioned to minimize damage to the samples. During tensile tests, each specimen was sprayed with phosphate buffering solution at ambient temperature to prevent dehydration and loaded until failure under quasistatic conditions at 5 mm/min displacement rate to avoid viscoelastic effects.^17^^,^^18^ The force and displacement were measured during the test. The elastic modulus, a material property related to stiffness, was then calculated as the slope of the calculated stress-strain curve in the physiologic range with stress estimated from the systolic and diastolic pressure recorded in a preoperative visit for patients with aneurysmal disease; blood pressure was assumed to be 120/80 mm Hg among normal donor controls without aneurysmal disease (Fig 1).

### Data analysis

The force (*F*)-deformation (*D*) curve obtained by the uniaxial tensile test was converted to a true stress-true strain curve. The true stress (σ_t_) and the true strain (*ε*_t_) were calculated with the engineering stress (σ_e_) and the engineering strain (*ε*_e_). The engineering stress was defined with force (*F*) and an initial cross-sectional area (*A*_0_):

(1) $$\sigma_{e}=\frac{F}{A_{0}}$$

The engineering strain (*ε*_e_) was defined with the initial length (*L*_0_) and the deformation of the specimen (*D*):

(2) $$\varepsilon_{e}=\frac{D}{L_{0}}$$

To obtain true stress and true strain, we assumed that the aortic wall is incompressible, and the actual dimension of the specimen is subsequently changed during deformation. The total volume (*V*) of the specimen is constant:

(3) $$V=AL=A_{0}L_{0}$$

The true stress was calculated with the engineering stress and the engineering strain:

(4) $$\sigma_{t}=\frac{F}{A}=\frac{F}{A_{0}}\times \frac{A_{0}}{A}=\sigma_{e}\times \frac{L}{L_{0}}=\sigma_{e}\times \frac{L_{0}+D}{L_{0}}=\sigma_{e}\left(1+\varepsilon_{e}\right)$$

The true strain (*ϵ*_t_) was calculated by integrating an instantaneous true strain (d*ϵ*_t_), which is defined with an initial length of the specimen (*L*_0_) and an instantaneous stretch (d*L*):

(5) $$\varepsilon_{t}=\int d\varepsilon_{e}=\int_{L_{0}}^{L}\frac{\mathrm{dL}}{L}\;=\frac{L}{L_{0}}=\ln\left(1+\varepsilon_{e}\right)$$

We note that stresses and strains were either circumferential (σ_e,θθ_, σ_t,θθ_, *ε*_e,θθ_, *ϵ*_t,θθ_) or longitudinal (σ_e,ll_, σ_t,ll_, *ϵ*_e,ll_, *ε*_t,ll_) and are noted as such throughout the text.

To calculate mechanical properties in the physiologic range of in vivo loading, stresses (σ) at systolic and diastolic pressure were estimated by applying Laplace's law below with the radius of aorta (*r*), the thickness of specimens (*t*), and the systolic and diastolic pressure (*p*) recorded in a preoperative visit for patients with aneurysmal disease; blood pressure was assumed to be 120/80 mm Hg among normal donor controls without aneurysmal disease.

(6) $$\sigma =\frac{rp}{ct},\;c=\left\{ \begin{array}{l} 1,\;circumferential\;stress\\ 2,\;longitudinal\;stress \end{array}\right.$$

Mean elastic modulus (*E*_M_) was obtained as the average slope in the systolic and diastolic stresses. Relative strain (ε_rel_) was calculated from the ratio of a strain at diastolic stress to a strain at systolic stress:

(7) εrel=εsystolicεdiastolic

**Fig 2:**
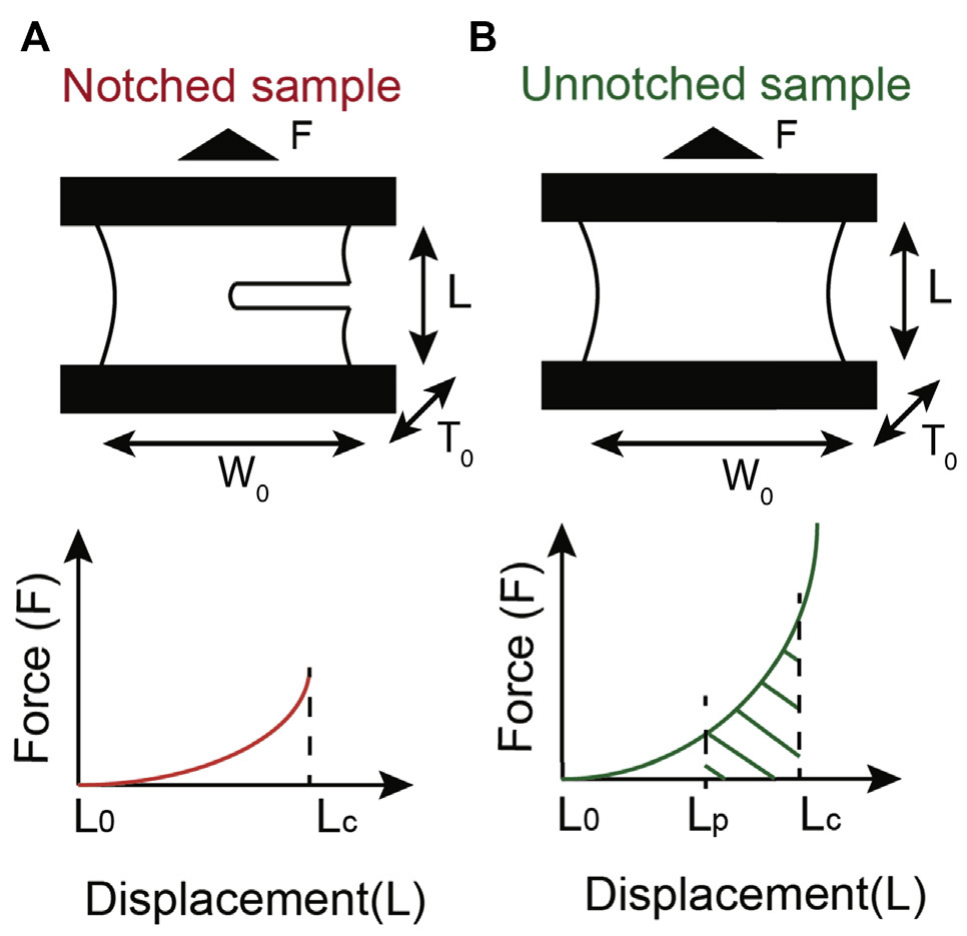
Experimental design for aortic tissue fracture toughness using notched **(A)** and unnotched **(B)** samples. *F*: force, *W*_0_: initial sample width, *T*_0_: initial sample thickness, *L*: sample length. *L*_0_: initial length, *L*_p_: physiologic length, *L*_c_: critical displacement (fracture toughness).

Fracture toughness of tissue samples was characterized with fracture energy measured with a notched fracture toughness test.^19^, ^20^, ^21^, ^22^, ^23^ As described in Fig 2, the notched fracture toughness test was conducted with two identical specimens prepared with the same width *W*_0_, thickness *T*_0_, and length *L*_0_. One sample was notched with a half-length of width (0.5*W*_0_) at the midway point and was stretched to critical displacement (*L*_c_) at which crack propagation started. The critical displacements (*L*_c_) were the measured displacements at which the initial kink of the force-deformation curve is started. The other sample was stretched following the same procedure of the uniaxial tension test. From two force-displacement curves of specimens, the fracture energy of the tissue sample was calculated by the following equation:

(8) Γ=∫LpLcFdlW0T0

Where *L*_p_ is the physiologic length.

### Aortic size measurements

Aortic size and growth rate for patients with aneurysmal disease were determined by obtaining measurements from the Stanford 3DQ lab (Stanford, CA), which routinely obtains orthogonal measurements using curved planar reconstructions in all patients undergoing surveillance for aortic diseases at Stanford Healthcare. Inner-to-inner wall aortic measurements were recorded at prespecified locations (aortic annulus, sinus of Valsalva, sinotubular junction, and mid ascending aorta), and the change over a given period of time was calculated. Changes <2 mm were attributed to measurement error and recorded as no change. The change in aortic size was then divided by time between scans to obtain an annualized rate of aortic growth. CT scans used were more than 60 days apart (mean, 302 days), and only patients with aneurysm with two or more available CT scans were included in the study.

For donor controls, routine CT used in the donor assessment protocol was reviewed to ensure that the aorta was not aneurysmal. In addition, the aorta was visually inspected at the time of organ recovery. However, orthogonal measurements could not be obtained because of the lack of appropriate software at outlying hospitals. Donor aortic size was estimated using a combination of age, sex, and body mass index based on normal measurements.^24^ Donor aortic growth rate was assumed to be 0 mm per year in control patients.

### Histology

After tissue recovery, aortic tissue was marked with a methylene blue pen to ensure accurate orientation (circumferential vs longitudinal) of the tissue. The tissue was then fixed in 10% formalin and embedded in paraffin. Aortic samples were stained with Accustain Elastin Verhoeff's Van Gieson kit (Sigma Aldrich, St. Louis, Mo) according to the manufacturer's instructions. Masson trichrome staining was performed with Bouin's solution and Weigert's iron hematoxylin solution (Sigma Aldrich, St. Louis, Mo). Representative sections both circumferentially and longitudinally were reviewed by a pathologist blinded to the underlying disease process (control, chronic dissection, or aneurysm). Specimens were graded qualitatively from 0 to 3 on mucoid extracellular matrix accumulation (MEMA), elastin fragmentation, medial fibrosis, and medionecrosis.^25^

### Statistical analysis

All analyses were performed in R 4.0.5. Our analysis began with assessing whether location (root, ascending, arch, or descending) and directionality (circumferential vs longitudinal) affected mechanical properties; the results of these comparisons would inform the remainder of our analysis. For comparisons among the various segments of aorta, a linear mixed-effects model was constructed with patient as a random intercept to account for the correlated nature of the data, that is, multiple sites (root, ascending, arch, descending), from the same patient as these measurements are not truly independent.

Mechanical properties were subsequently compared between normal controls and patients with either aneurysmal disease or chronic aortic dissection. In addition, histology was also compared between normal controls and patients with either aneurysmal disease or chronic dissection. A linear mixed-effects model was constructed with patient as a random intercept to account for the correlated nature of the data.

Multiple machine learning techniques were used to develop a predictive model for aortic growth rate: multiple imputation with predictive mean matching, modified bootstrap model selection, and Least Absolute Shrinkage and Selection Operator (LASSO). Each technique will be discussed in detail below. The added value of machine learning techniques over traditional statistical methodology is the improved ability to predict and also the ability to handle situations in which the number of predictors is large, including when the number of predictors is larger than the number of observations, for example, genome-wide association studies.

Our model construction was carried out in two stages: first we performed variable selection, and this was followed by coefficient estimation. Using the average value for each aortic segment for the individual patients, multiple imputation with predictive mean matching was used to create 20 imputed datasets using the *mice* package. Aortic root samples are smaller than ascending samples; thus the number of tests that can be performed is lower. Therefore, multiply imputed data allowed us to avoid listwise deletion of incomplete observations without affecting the variance. LASSO—a standard technique for creating a sparse predictive model in the setting of a large number of predictors^26^ on linear regression—was performed using the *glmnet* package in each of the 20 imputed datasets to create a set of parsimonious models; the lambda penalty was obtained by cross-validation in each individual model. Final variable selection was then performed by selecting only the variables that appeared in at least 60% of the models (at least 12 of the 20 imputed datasets) and with consistent sign for the beta coefficient (>80% either positive or negative); this process is akin to the methodology used for bootstrap variable selection to improve the performance of automatic selection techniques.^27^ After variable selection with the machine learning techniques described above, a pooled linear mixed-effects model with patient as a random intercept was used to evaluate aortic growth rate as a function of these variables—including anatomical location as a categorical variable to account for differences between anatomical sites. Circumferential and longitudinal direction and relative strain were separately assessed under the supposition that relative strain and directionality are separate stable effects.

The potential association between histologic variables and mechanical properties was explored using linear regression. Model selection was performed using backward selection in 100 bootstrap replicates. Use of bootstrapping reduces the potential for spurious associations seen with automatic selection techniques in isolation.^27^ The analysis was performed using the *bootstepAIC* package and adjusted for subject effect within the mixed-effects model.

The two-tailed *P* value <.05 was considered to be statistically significant. Because of the exploratory nature of this analysis, no adjustment for multiple comparisons was performed.^28^

## Results

Multiple samples from 31 patients were analyzed (3 ± 0.9 per patient and aortic segment). In total, there were 405 samples measured for stiffness, 419 samples measured for toughness, and 412 samples measured for relative strain. The distribution of patient types was as follows: 14 healthy organ donors, 3 healthy organ recipients, 6 patients with chronic aortic dissection with aneurysmal degeneration, and 8 patients with intact aortic aneurysms. There were 4 patients with Marfan syndrome, 1 patient with Loeys-Dietz syndrome, and 1 patient with bicuspid aortic valve (Table I). The mean age of patients in our cohort was 46.7 years. With respect to sample location, there were 11 aortic root specimens, 14 for the ascending aorta, 14 for the aortic arch, and 18 for the descending thoracic aorta (Table I).

**Table I:** Demographic information and aortic disease classification of tissue donors (n = 31)

	n = 31
Age, mean (SD)	46.7 (17.1)
Male, n (%)	21 (67.7)
Race, n (%)	
Asian	2 (6.5)
Asian/Pacific Islander	4 (12.9)
White	22 (71.0)
Not recorded	3 (9.7)
Ethnicity, n (%)	
Hispanic	9 (29.0)
Not Hispanic	19 (61.3)
Not recorded	3 (9.7)
Weight, mean (SD)	86.13 (23.04)
Location, n (%)	
Root	11 (19.3)
Ascending	14 (24.6)
Arch	14 (24.6)
Descending	18 (31.6)
Classification, n (%)	
Aneurysm	8 (25.8)
Chronic dissection	6 (19.4)
Donor	14 (45.2)
Recipient	3 (9.7)
Connective tissue disease, n (%)	
Bicuspid aortic valve	1 (3.2)
Loeys-Dietz	1 (3.2)
Marfan syndrome	4 (12.9)
None	25 (80.6)

*SD*, Standard deviation.

### Location (root, ascending, arch, descending) and direction (circumferential vs longitudinal) influence mechanical properties in the normal aorta

**Fig 3:**
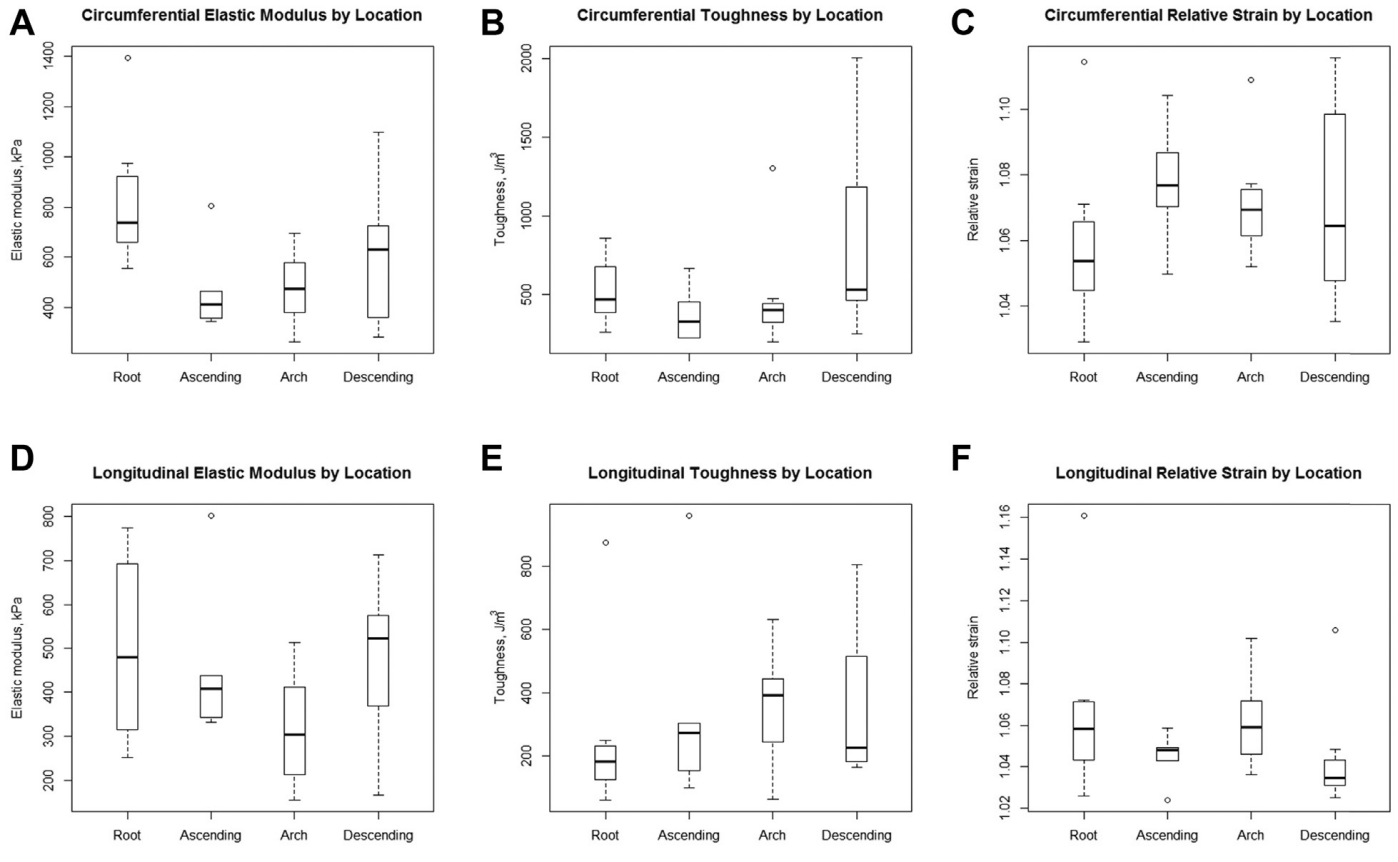
Aortic tissue mechanical properties by aortic segment. Aortic segment location (root, ascending, arch, or descending thoracic aorta) affected elastic modulus (**A** and **D**), toughness (**B** and **E**), and relative strain (**C** and **F**) in the circumferential and longitudinal directions. The *box* denotes upper and lower quartiles, the *solid horizontal line* represents the median, and *whiskers* represent ±1.5 interquartile range.

We examined 17 patients with normal aortas (either organ donors or organ recipients) with samples from the aortic root (8), ascending aorta (6), aortic arch (11), and descending thoracic aorta (12). Location, that is, aortic segment, significantly affected mean elastic modulus, toughness, and relative strain in both the circumferential and longitudinal directions at physiologic pressures, that is, 120/80 mm Hg, and this suggested that our analysis may have to account for differences in mechanical properties among the various segments of the aorta (Fig 3).

**Fig 4:**
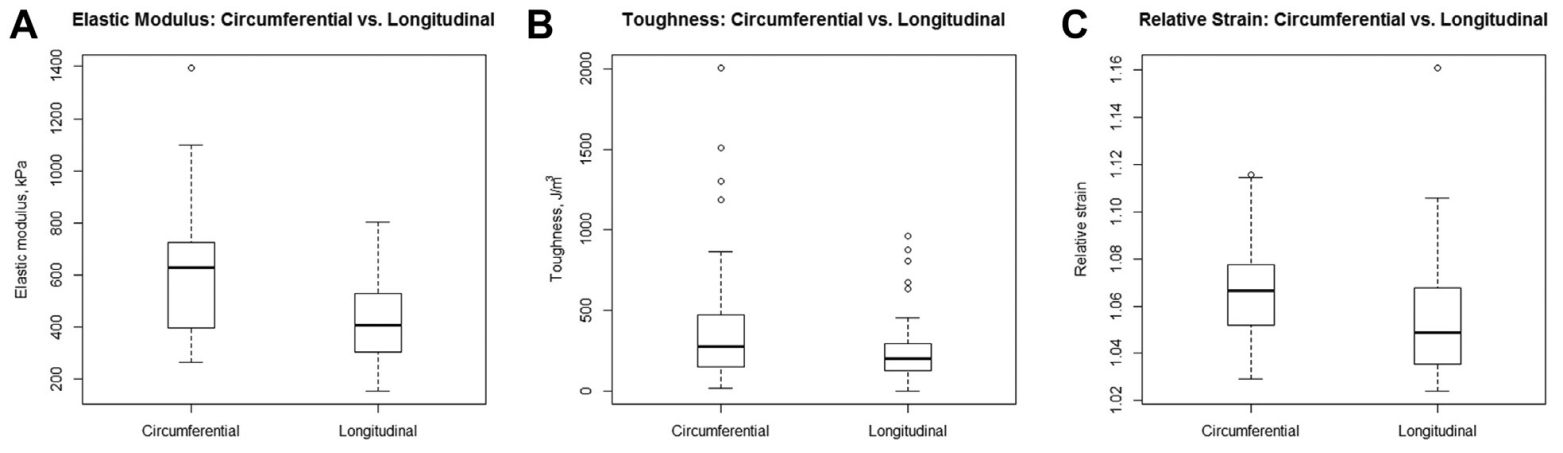
Comparison of circumferential and longitudinal aortic mechanical properties. The longitudinal direction had lower elastic modulus **(A)**, was less tough **(B)**, and experienced less relative strain **(C)** as compared with the circumferential direction after accounting for location and using a repeated measures technique to account for correlated data. The *box* denotes upper and lower quartiles, the *solid horizontal line* represents the median, and *whiskers* represent ±1.5 interquartile range.

Comparing the circumferential and longitudinal directions (Table II), longitudinal elastic modulus was less than circumferential elastic modulus after accounting for specimen location: −166.8 kPa (95% confidence interval [CI]: −210.8 to −122.7, *P* < .001). Fracture toughness was also less in the longitudinal direction than in the circumferential direction after accounting for specimen location: −201.2 J/m^3^ (95% CI: −272.9 to −129.5, *P* < .001). Finally, relative strain was less in the longitudinal direction than in the circumferential direction after accounting for specimen location: −0.01 (95% CI: −0.02 to −0.004, *P* = .002; Fig 4). This suggested that directionality of measurement may also have to be accounted for in the remainder of our analysis.

**Table II:** There were statistically significant differences in the mean elastic modulus, toughness, and relative strain among the different aortic segments of the normal aorta compared with the root as a reference (Ref)

	Elastic modulus, kPa			Toughness, J/m^3^			Relative strain		
	Est.	95% confidence interval	*P* value	Est.	95% confidence interval	*P* value	Est.	95% confidence interval	*P* value
Circumferential									
Root	Ref	Ref	Ref	Ref	Ref	Ref	Ref	Ref	Ref
Ascending	−303.0	−430.4 to −175.5	**<.001**	−88.7	−282.4 to 104.9	.4	0.012	−0.004 to 0.028	.2
Arch	−445.1	−576.9 to −313.3	**<.001**	−129.9	−339.7 to 79.8	.2	0.023	0.006 to 0.040	**.008**
Descending	−313.6	−440.1 to −187.0	**<.001**	77.0	−122.3 to 276.3	.5	0.020	0.004 to 0.036	**.01**
Longitudinal									
Root	Ref	Ref	Ref	Ref	Ref	Ref	Ref	Ref	Ref
Ascending	−34.5	−139.7 to 70.8	.5	205.7	59.9 to 351.5	**.007**	−0.021	−0.041 to −0.001	**.04**
Arch	−110.6	−215.4 to −5.8	**.04**	120.7	−33.5 to 275.0	.1	−0.015	−0.035 to 0.005	.1
Descending	−42.4	−147.4 to 62.5	.4	−0.1	−154.6 to 154.5	.1	−0.026	−0.046 to −0.006	**.01**

For circumferential toughness, statistically significant differences arose when comparing aortic segments with the descending thoracic aorta as the reference, that is, differences were significant when comparing the descending with the ascending and arch.

Boldface *P* values represent statistical significance.

### Disease state affects toughness and relative strain but not elastic modulus

**Fig 5:**
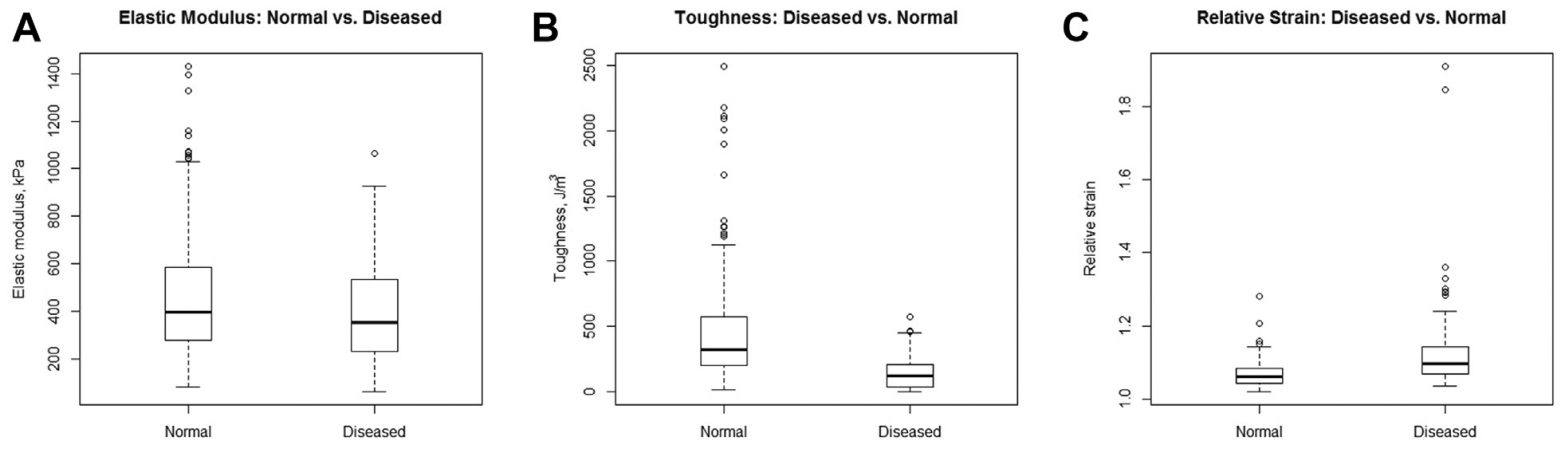
Comparison of normal and diseased aortic mechanical properties. In comparison with normal aorta, patients with chronic dissection or aneurysmal disease had similar elastic modulus **(A)** but were less tough **(B)**. Specimens from patients with chronic dissection or aneurysmal disease experienced significantly greater relative strain **(C)** than our normal controls. The *box* denotes upper and lower quartiles, the *solid horizontal line* represents the median, and *whiskers* represent ±1.5 interquartile range.

When comparing normal aorta with diseased aorta—that is, either aneurysmal or chronic dissection—after accounting for location and direction, disease state did not affect mean elastic modulus, −94.8 kPa (95% CI: −202.7 to 13.2, *P* = .1). However, patients with diseased aorta had decreased toughness as compared with normal aorta, −431.7 J/m^3^ (95% CI: −628.6 to −234.8, *P* < .001). Finally, diseased aorta experienced increased relative strain, 0.09 (95% CI: 0.04 to 0.14, *P* = .003; Fig 5).

### Mechanical properties of the aorta predict aortic growth rate

Using our machine learning technique, sex, aortic size (radius), aortic segment (root, ascending, arch, or descending), classification of disease (normal, aneurysm, or chronic dissection), relative strain, toughness, and age were found to be consistent predictors of aortic growth over time (Table III). The ascending, arch, and descending were all more apt to grow than the aortic root. In addition, chronic aortic dissection was a statistically significant independent predictor of aortic growth. These findings appear to be in line with current published guidelines, which differentiate among ascending, arch, and descending aneurysms in addition to providing different thresholds to operate for nondissected descending thoracic aortic aneurysms and chronic dissections.^29^ The difference in the predilection to grow identified in our analysis may underlie the differences in natural history observed among patients with different sized aneurysms at the time of diagnosis in the landmark study published by the Yale group.^2^

**Table III:** Predictive model for aortic growth (mm/y)

	Estimate	95% confidence interval	*P* value
Intercept	−8.88	−14.348 to −3.412	**.002**
Female sex	3.07	−0.497 to 6.637	.09
Radius, mm	0.40	0.341 to 0.459	**<.001**
Aortic segment			
Root	Ref	Ref	Ref
Ascending	1.34	1.183 to 1.497	**<.001**
Arch	1.81	1.594 to 2.026	**<.001**
Descending	2.45	2.156 to 2.744	**<.001**
Aortic pathology			
No aneurysm	Ref	Ref	Ref
Aneurysm	−0.97	−5.243 to 3.303	.7
Chronic dissection	6.97	2.815 to 11.125	**.001**
Relative strain	3.64	1.445 to 5.835	**.005**
Toughness, J/m^3^	0.00	0.0 to 0.0	.8
Age, y	−0.08	−0.178 to 0.018	.1

Increasing aortic size and circumferential relative strain were independent predictors of aortic growth rate over time. The arch and descending aorta were more apt to grow than the aortic root. The ascending aorta was no different from the aortic root.

Boldface *P* values represent statistically significant independent contributions of the variable.

Notably, higher relative strain was a statistically significant predictor of aortic growth over time after controlling for other variables, and this finding has not been previously described in the literature. Despite being consistent predictors, the remaining variables (sex, toughness, and patient age) failed to be statistically significant in the pooled linear mixed-effects model (Table III). Other variables—mean elastic modulus, direction (circumferential vs longitudinal), presence or absence of connective tissue disease—failed to be consistent predictors in our model selection technique.

### Aortic histopathology correlates with diseased state and mechanical properties

**Fig 6:**
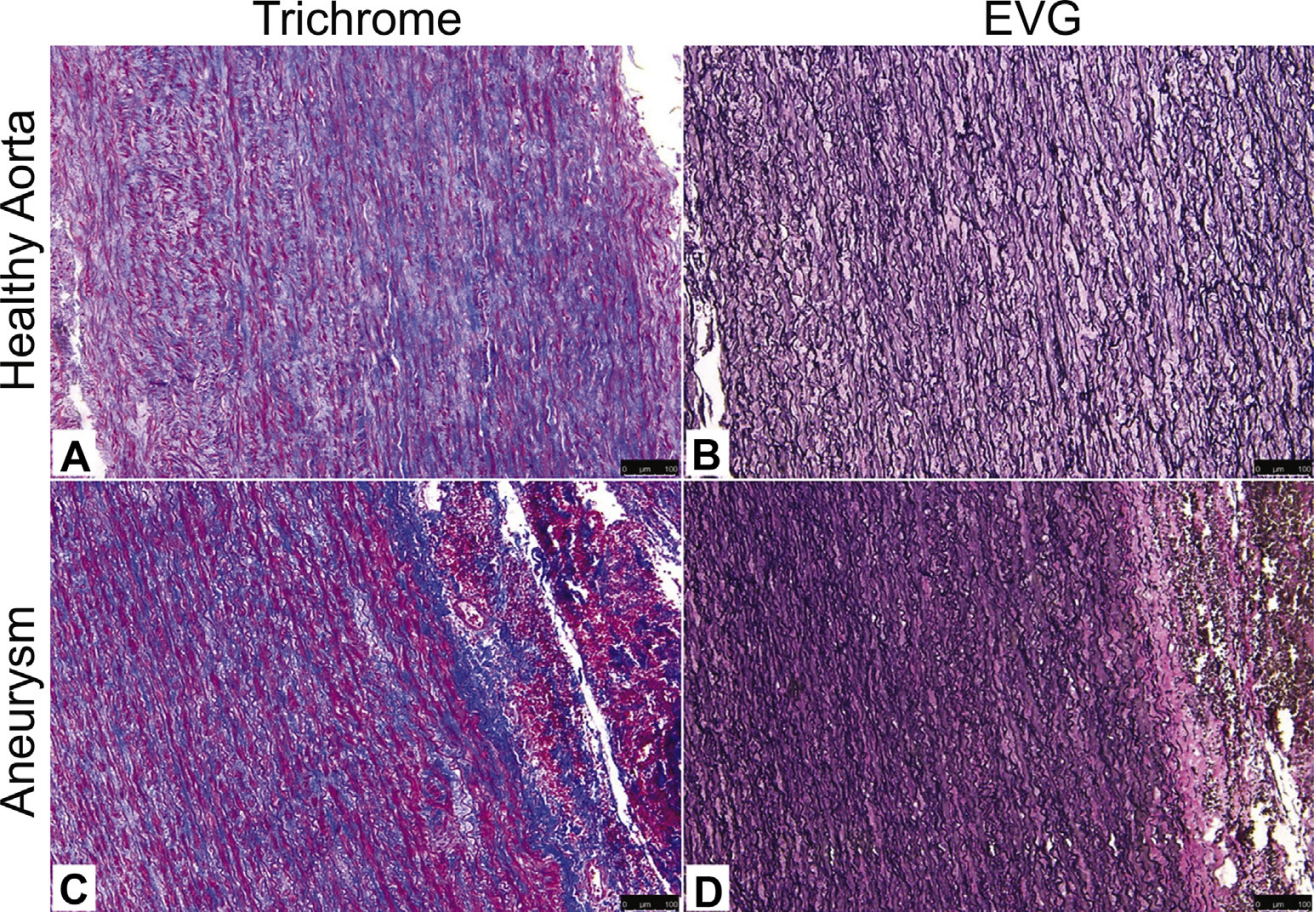
Representative images of ascending aorta with trichrome and EVG stains. Healthy donor ascending aorta: trichrome **(A)**, healthy donor ascending aorta: EVG **(B)**, bicuspid aortic valve, ascending aneurysm: trichrome **(C)**, bicuspid aortic valve, ascending aneurysm: EVG **(D)**. *EVG,* Elastin van Gieson.

**Fig 7:**
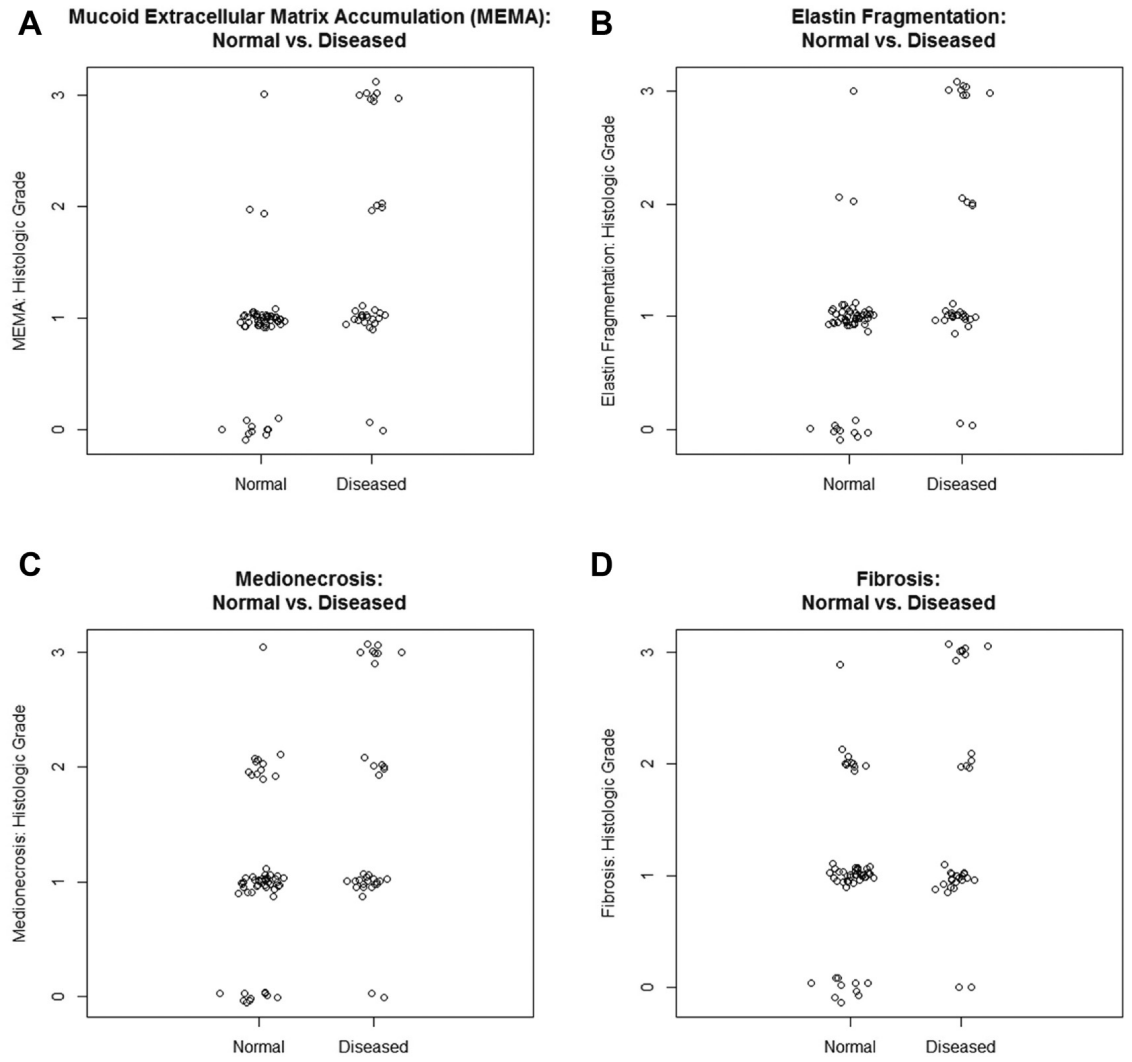
Quantification of histologic differences in MEMA **(A)**, elastin fragmentation **(B)**, medionecrosis **(C)**, and fibrosis **(D)**. Chronic dissection or aneurysmal disease tended to have higher histological grades in all categories compared with normal controls. MEMA (0.8 ± 0.2, *P* = .001), elastin fragmentation (0.8 ± 0.2, *P* = .001), medionecrosis (0.7 ± 0.2, *P* = .01), and fibrosis (0.7 ± 0.2, *P* = .008).

Following review of specimens by a pathologist blinded to the patient history, aortic segment, and directionality of the specimens, histologic grades for MEMA, elastin fragmentation, medial fibrosis, and medionecrosis were compared. Representative slides from the patients are depicted in Fig 6. Patients with aortic pathology tended to have a higher histologic grade for MEMA (0.8 ± 0.2, *P* = .001), elastin fragmentation (0.8 ± 0.2, *P* = .001), medionecrosis (0.7 ± 0.2, *P* = .01), and fibrosis (0.7 ± 0.2, *P* = .008; Fig 7). Increasing fibrosis was associated with greater mean elastic modulus (180.0 kPa, 95% CI: 48.6 to 311.3, *P* = .009) after accounting for location and direction. In contrast, histology failed to correlate with either toughness or relative strain.

## Discussion

The current methods for assessing the risk for aortic catastrophe are limited to the presence or absence of a genetic syndrome such as Marfan or Loeys-Dietz syndrome, aortic segment, aortic diameter, and interval growth as assessed by serial CT. However, despite the existence of guidelines for intervention on the aorta, aortic catastrophe may occur at sizes smaller than the threshold for intervention.^4^^,^^30^ Furthermore, determination of aortic growth requires multiple measurements over time. Given the risks associated with watchful waiting, determining predictors of aortic growth may be of significant benefit for identification of patients who have not yet reached the size criteria required for intervention on the aorta but may still be at substantial risk for progression of disease, thus using a precision medicine-based approach. Given the development of novel techniques for the assessment of aortic biomechanics using noninvasive imaging techniques, such as transesophageal speckle-tracking to calculate the cardiac cycle stress and pressure modulus,^9^ we sought to investigate whether an association between aortic biomechanics and aneurysmal growth existed.

Our finding that the aortic root was stiffer than the remainder of the aorta was consistent with the report by Azadani et al,^31^ demonstrating that the aortic root had greater elastic modulus than the ascending aorta in organ donors. Differences in aortic compliance and elastic modulus have been shown to vary along the length of the aorta by Haskett et al,^32^ though the elastic modulus measured in their report increased along the length of the aorta from proximal to distal and varied with respect to age. The differences between this report and ours may have been related to the method for preparation and harvest. The samples used by Haskett et al were primarily retrieved from the autopsy suite and tested within 36 hours of retrieval; however, the duration of time between patient death and autopsy was not reported. Stemper et al^33^ and Chow et al^13^ independently demonstrated that prolonged cold storage may result in alterations in the mechanical properties of the tissue, and this introduces the possibility of tissue degradation between the time of patient death and mechanical testing. In contrast, our samples were all tested within 24 hours of application of the aortic cross clamp, that is, cessation of blood flow to the aorta, and tested fresh leading to minimal degradation.

In our cohort, the mechanical properties of the aorta differed between the longitudinal and circumferential directions with respect to elastic modulus, fracture toughness, and relative strain at in vivo physiologic pressures. We note that the mechanical stresses resulting from systolic and diastolic pressure that were used to calculate the mean elastic modulus were different for the case of the longitudinal direction vs circumferential direction to simulate the case of in vivo loading, where the circumferential stress is expected to be twice as high as the longitudinal stress for any given pressure. In each case, the longitudinal direction was measured to be less than the circumferential direction. Whereas this finding has previously been demonstrated with respect to elastic modulus,^5^^,^^7^ that fracture toughness and relative strain also differed between the circumferential and longitudinal directions appears to be a novel finding. Whereas relative circumferential or longitudinal strain in this study was assessed using the unloaded reference configuration to calculate the initial systolic and diastolic strains, in vivo cyclic strain could be measured with the use of end-diastole as the smallest reference configuration.

Whether increasing collagen deposition, which has been associated with increasing aortic elastic modulus in the literature,^34^ is an etiologic factor or merely a sequela of aneurysmal degeneration is uncertain; the nature of this relationship would determine whether it is a protective or pathologic process. The finding that higher relative strain in physiologic conditions was predictive of aneurysmal progression may suggest that increasing fibrosis leading to increased elastic modulus is an adaptive—rather than maladaptive—reaction to aneurysmal progression as increasing elastic modulus may serve to limit distensibility and ultimately dilation. This interpretation remains somewhat speculative as fibrosis was not predictive of relative strain in our analysis. Pichamuthu et al^5^ have been able to demonstrate that collagen organization and aortic orientation contributed to differences in aortic tensile strength and strain in the ascending aorta among patients with bicuspid and tricuspid aortic valves. The results of their report may offer a pathologic mechanism for the association between strain and aortic growth rate that we found, that is, that increased distensibility is indicative of a failure to adequately reduce wall stress through extracellular maxtrix collagen remodeling. This phenomenon has been demonstrated in the abdominal aorta but has not been shown in the thoracic aorta.^35^

Interestingly, this reduction in strain has been observed in the aging aorta.^36^^,^^37^ However, age failed to be a consistent predictor when using our machine-learning variable selection technique. This may have been due to the small number of patients in our study population. Indeed, with age-related baseline differences in strain observed in the literature thresholds for relative strain may need to be indexed to age.

With the development of electrocardiographically gated multidetector CT scan technology, it is possible to measure minute differences in aortic distention throughout the cardiac cycle^10^; therefore, incorporation of relative strain into the routine assessment of the aorta may be both feasible and informative. A prospective cohort study correlating aortic distensibility and relative strain measurements with aortic growth rate using four-dimensional CT angiography may be able to both provide age-indexed normal values and validate the association discovered in the current report.

This study was limited by its retrospective rather than prospective assessment of aortic growth. Given that the specimens were excised at the time of operation, assessment of aortic histopathology and biomechanical properties occurred only after the development of disease meeting surgical criteria and thus were ex post facto, as it were. Whether this relationship would hold prospectively would require a study in a larger observational cohort, which would also allow for adjustment in the setting of multicollinearity between age and aortic strain.

In total, there were 31 patients contributing over 400 samples to each of our analyses. However, the large number of potential predictors necessitated the use of a machine learning technique, LASSO, to create a sparse model, that is, a model with a limited number of terms. Limitations of LASSO include an inability to differentiate between grouped variables, that is, multiple variables that are closely related. In addition, in the case where the number of predictors is greater than the number of observations, the model may saturate with the largest number of predictors being the same as the number of observations due to the process of optimization.^38^ In this instance, though, the number of observations was greater than the number of predictors. Random intercepts may not account for sample replicates, and alpha inflation may be present due to a limited sample size but is consistent across each disease condition.

Lastly, our use of uniaxial loading simplifies the mechanical testing but reveals directional differences of the tissue mechanical properties between conditions. The mean elastic modulus in the physiologic range of the in vivo loading state serves as a convenient metric to compare the mechanical properties between conditions or directions. However, like other collagen-rich biological tissues, the aorta is a nonlinear viscoelastic material, and our measurements and analyses do not fully capture the mechanical response to higher strain rates with viscoelastic response that the aorta experiences in vivo. Furthermore, the biomechanical testing was performed ex vivo without external tethering or support. Recreating all in vivo mechanical parameters is nearly impossible during ex vivo mechanical testing. Nonetheless, the comparison between samples is still meaningful because all testing was performed under the same conditions.

## Conclusions

Assessing the risk of rupture or dissection for thoracic aortic aneurysms remains an imperfect science with the only criteria for surgical intervention being the presence of a genetic syndrome, aortic segment, aortic diameter, and growth over time. However, these may be inadequate for the prediction of aortic catastrophe as dissection and rupture may occur in patients not yet meeting criteria for intervention. We found that relative strain was a novel predictor of aneurysmal progression after accounting for sex, aortic diameter, location, and disease state. Use of this metric in vivo may be a feasible and informative adjunct in the routine surveillance of thoracic aortic aneurysms, though its application requires additional study in collaboration with our radiology colleagues.

## Author contributions

Conception and design: PC, HL, OC, MF

Analysis and interpretation: PC, HL, AD, AC, OC, MF

Data collection: PC, HL, TK, MN, AC

Writing the article: PC, HL, AD, TK, MN, AC, OC, MF

Critical revision of the article: PC, HL, AD, OC, MF

Final approval of the article: PC, HL, AD, TK, MN, AC, OC, MF

Statistical analysis: PC, HL

Obtained funding: Not applicable

Overall responsibility: MF

PC and HL contributed equally to the work.
